# Catenane organocatalyst breaks the linear scaling limitation through conformational selection

**DOI:** 10.1038/s42004-023-01088-w

**Published:** 2024-01-08

**Authors:** Chenyu Wang

**Affiliations:** Scientific Reports, https://www.nature.com/srep/

**Keywords:** Interlocked molecules, Homogeneous catalysis, Organic chemistry

## Abstract

Conformational dynamics are integral to enzyme catalysis, yet they are barely explored when designing synthetic catalysts. Now, a catenane-based organocatalyst, which dynamically switches between two conformations, speeds up the catalysis of carbodiimide hydration through spontaneous conformational adaptation for different reaction steps.

Traditional catalysts with static configurations often grapple with the linear scaling barrier: enhancing the efficiency of one reaction step can inadvertently decelerate another. This challenge lies not just with the complexity of the reactions but also in the inherent limitations of synthetic catalysts, which typically remain fixed in their conformations, unable to adapt to the dynamic environment. This rigidity results in enhancements in a catalyst’s property only yielding proportional improvements, rendering significant leaps in efficiency hard to achieve.

Now, inspired by biological molecular motors that naturally adopt different conformations for different tasks^1^, Prof David Leigh from the University of Manchester, UK and colleagues have designed a catenane-based organocatalyst that changes its conformation in order to selectively accelerate different reaction steps at different stages of a catalytic process, thereby overcoming the linear scaling limitation (10.1016/j.chempr.2023.10.019)^2^.

The catalysis of carbodiimide hydration by the catenane-based organocatalyst involves ester formation and ester hydrolysis, which proceed at varying rates contingent on the catalyst’s conformation. Figure 1 illustrates the variation of the overall reaction rate as a function of the catalyst’s structure, with which either ester formation or ester hydrolysis becomes the rate-limiting step. Specifically, the catenane’s proximal conformation promotes ester hydrolysis via cooperative hydrogen bonding, while the distal conformation, with a less sterically hindered carboxylate group, favorably speeds up ester formation. The shift in equilibrium of the conformational states, triggered by the variation of bound reactants, enables the catalyst to adopt the preferable conformation at different stages of the catalytic cycle. As a result, both ester formation and ester hydrolysis steps are substantially accelerated. Indeed, the team found that the dynamic organocatalyst displayed activity of up to 10 times faster than that of any static counterpart, and showed that the accelerated rates observed matched the accelerations achieved in the catalytic steps that were sped up by conformational selection.

**Fig. 1:**
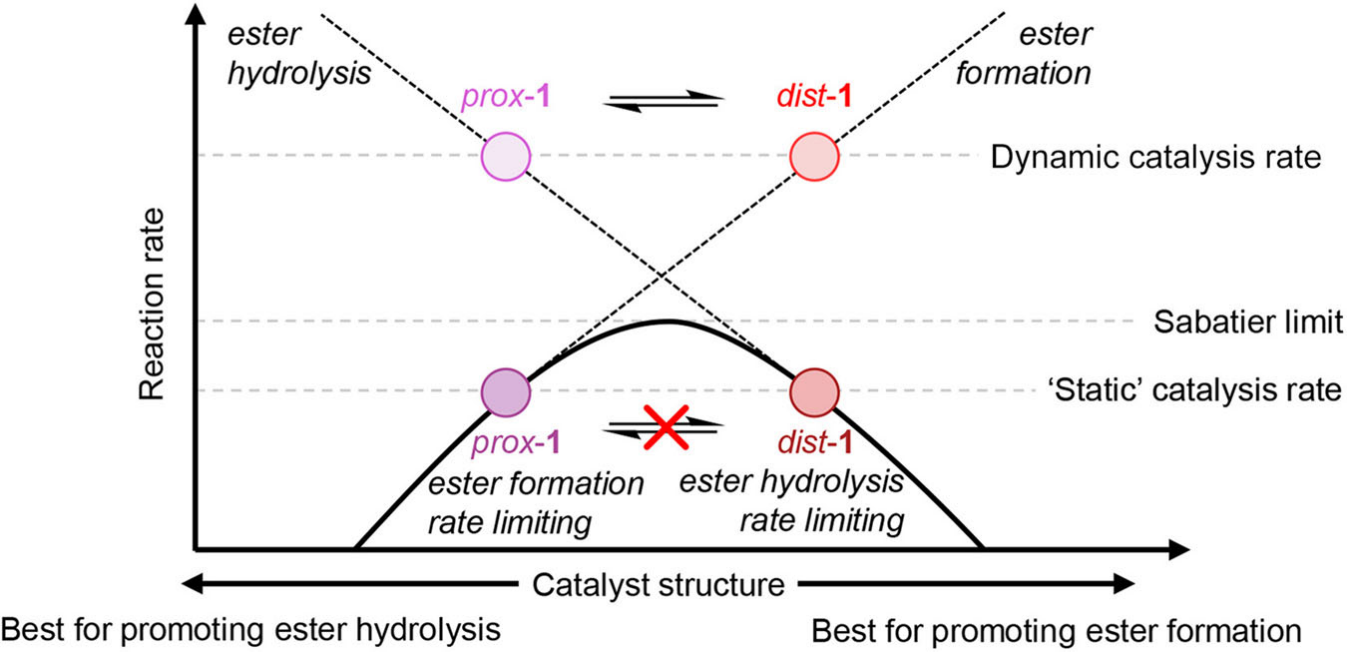
Dynamically accelerated catalysis. Volcano plot showing how catalysis by the catenane-based organocatalyst with dynamically interchangeable conformations goes beyond the linear scaling limitation. Adapted from Chem 10.1016/j.chempr.2023.10.019. Copyright (2023) Elsevier.

Concomitant with its catalytic activity, the organocatalyst was also found to display directionally biased rotation, reminiscent of natural biomolecular motors performing catalysis and mechanical tasks in the energy conversion cycle. “The link between faster catalysis and directionally biased motion may imply that motor molecules, essential components of cellular processes, could have originally arisen as a consequence of the evolutionary pressure to more efficiently convert chemical energy into mechanical work for a faster self-replication,” suggests Leigh.

Looking to the future, the researchers will continue leveraging design principles for molecular motors to develop other artificial catalysts, aiming to relieve more catalytic reactions from the constraints of the linear scaling limitation. “After all, biology uses the ratcheting of molecular motors that occurs through catalysis to achieve function in every biological process, from force generation to transport to synthesis. Surely, synthetic chemists can do the same,” concludes Leigh.
